# Patient Perceptions of Robotic-Assisted Hip and Knee Arthroplasty Among Orthopaedic Outpatient Attendees: A Cross-Sectional Survey in an Irish Tertiary Center

**DOI:** 10.1016/j.artd.2026.102062

**Published:** 2026-06-04

**Authors:** Conor Farrell, Orla Hennessy, Dunamis Akinyemi, Angela Faustino, Fiachra Rowan, May S. Cleary

**Affiliations:** a Waterford University Hospital, Waterford, Ireland; b Royal College of Surgeons in Ireland, Dublin, Ireland; c Department of Orthopaedic Surgery, University College Cork, Cork, Ireland

**Keywords:** Robotic-assisted arthroplasty, Patient perceptions, Hip arthroplasty, Knee arthroplasty, Shared decision-making

## Abstract

**Background:**

Robotic-assisted arthroplasty has been introduced to improve the precision and reproducibility of implant positioning in joint replacement surgery. While clinical and economic evaluations continue to expand, little is known about patient perceptions of this technology, and no Irish data currently exist. This study evaluates patient awareness, attitudes, and expectations regarding robotic-assisted joint replacement in an Irish tertiary orthopaedic center.

**Methods:**

A cross-sectional paper-based survey was administered to adult patients attending elective orthopaedic outpatient clinics between August and November 2025. The questionnaire assessed awareness, perceived risks and benefits, willingness to undergo robotic-assisted surgery, and views on surgeon vs robot control using yes/no questions and 0–10 Likert scales. Descriptive and comparative analyses were performed.

**Results:**

A total of 117 patients participated. Awareness of robotic-assisted arthroplasty was modest, with 38.5% reporting prior knowledge of the technology. Most patients (87.2%) wished to be informed if a robot were involved in their operation, yet only 11.1% would change surgeons to access robotic-assisted surgery. Awareness significantly influenced expectations: those who had heard of robotics anticipated better outcomes (6.5 ± 1.8 vs 5.0 ± 2.1, *P* < .001), less postoperative pain (4.8 ± 1.5 vs 5.7 ± 1.8, *P* = .006), and lower risk (4.7 ± 2.1 vs 5.8 ± 2.3, *P* = .019) than those unaware. Perceptions of invasiveness, operative time, cost, recovery, and robot independence did not significantly differ. Age did not influence awareness or perception across any domain.

**Conclusions:**

This first Irish study demonstrates low awareness and mixed optimism toward robotic arthroplasty. Prior awareness is associated with more favorable expectations, while trust in the surgeon remains central.

## Introduction

Total hip arthroplasty and total knee arthroplasty are among the most common and cost-intensive orthopaedic procedures worldwide, with utilization projected to rise substantially in the coming decades [1,2]. While both operations have been shown to be effective at reducing pain and improving function, a proportion of patients remain dissatisfied, citing residual pain, instability, stiffness or functional limitations [3,4]. Malalignment, suboptimal implant positioning and variability in soft tissue balance have been linked to complications and revision surgery [5,6].

Robotic-assisted arthroplasty has emerged as a technological advancement intended to improve the precision and reproducibility of implant placement in both the hip and knee [7,8]. Early studies report potential benefits in radiographic accuracy and short-term outcomes, although long-term superiority remains to be proven [8,9]. The introduction of robotic systems is also associated with increased costs, additional operative time, and a learning curve for surgeons [8].

Despite the focus on clinical and economic outcomes, less is known about patient perceptions of robotic-assisted joint replacement. Evidence from other surgical specialties suggests that while patients often associate robotic surgery with greater precision and better outcomes, awareness is generally low and misconceptions are common—particularly the belief that the robot may operate autonomously [10]. Recent work in arthroplasty demonstrates similar trends, with many patients reporting uncertainty about the benefits of robotic joint replacement, concerns about incision length, and little inclination to preferentially seek a robotic surgeon [11].

These findings highlight the need for specialty-specific evaluation to understand how patients perceive robotic technology within arthroplasty.

To date, no study has evaluated patient opinions on robotic-assisted hip and knee arthroplasty in the Irish healthcare setting. Understanding patient awareness, attitudes, and expectations is critical to inform consenting processes, shared decision-making, and future service planning.

## Methods

### Study design and setting

We conducted a cross-sectional survey of patients attending an elective orthopaedic outpatient clinic in a tertiary referral center between August and November, 2025. The study was designed to evaluate patient awareness, perceptions, and attitudes toward robotic-assisted hip and knee arthroplasty. Participation was voluntary and anonymous, and consent was implied by completion of the survey.

### Participants

A cross-sectional paper-based survey was administered to adult patients attending an elective orthopaedic outpatient clinic between August and November 2025, including patients being assessed for hip or knee pathology and potential arthroplasty. Patients under the age of 18 or those who were unable to complete the questionnaire due to cognitive impairment or language barriers were excluded. Consecutive patients were approached in clinic waiting areas by a member of the research team and invited to participate.

### Survey instrument

A paper-based questionnaire was developed to assess patient awareness, perceptions, and attitudes toward robotic-assisted hip and knee arthroplasty. The survey included demographic items (age, gender) and a series of yes/no and 0–10 Likert-scale questions. Items addressed awareness of robotic technology, willingness to undergo robotic-assisted surgery, perceived risks and benefits (including operative results, invasiveness, risk, operative time, pain, and recovery), perceived cost, and beliefs regarding the degree of surgeon vs robot control. The questionnaire was designed to be completed in 5–10 minutes (Appendix).

### Data collection

Consecutive adult patients attending an elective orthopaedic outpatient clinic during the study period were invited to complete a paper-based questionnaire in the clinic waiting area. Patients aged under 18 years were excluded. Patients unable to complete the questionnaire due to cognitive impairment or language barriers were also excluded at the point of survey administration. To avoid duplicate responses, patients were asked to complete the questionnaire only once during the study period. Exclusion due to cognitive impairment or language barriers was applied at the point of survey administration; however, these cases were not formally recorded and are therefore not included in the numerical breakdown. Clinic attendance records were retrospectively reviewed to determine the total number of patients attending during the study period and to define the eligible study population. This enabled calculation of the response rate and construction of a participant flow diagram in accordance with Strengthening the Reporting of Observational Studies in Epidemiology (STROBE) guidelines. A total of 269 clinic attendances were recorded. Of these, 31 patients were aged under 18 years and excluded. A further 12 duplicate attendances were identified and excluded, resulting in 226 eligible adult patients. Of these, 117 completed the questionnaire, yielding a response rate of 51.8%.

### Statistical analysis

All data were anonymized and analyzed using Microsoft Excel (Microsoft Corporation, Redmond, WA, USA). Descriptive statistics were calculated for all variables. Categorical data were summarized as frequencies and percentages, while continuous variables from the 0–10 Likert scales were summarized as mean ± standard deviation and treated as approximately interval-level data, consistent with common practice in survey-based research. Comparisons between independent groups (eg, those aware vs unaware of robotic-assisted surgery; <60 vs ≥ 60 years) were performed using independent-samples *t*-tests. Where equality of variances could not be assumed, Welch’s correction was applied. Chi-square tests were used to compare categorical variables. A *P* value of <0.05 was considered statistically significant. Missing data were handled using pairwise deletion.

## Results

**Figure 1:**
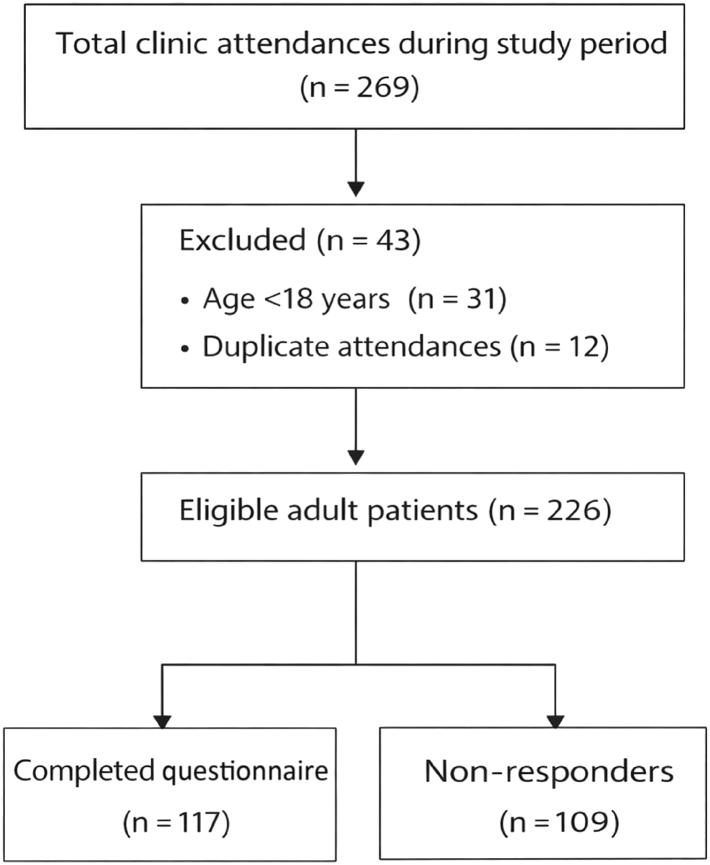
Flow diagram of participant recruitment and questionnaire response rates.

During the study period, 269 clinic attendances were recorded. After exclusion of patients aged under 18 years (n = 31) and duplicate attendances (n = 12), 226 eligible adult patients were identified. Of these, 117 completed the questionnaire, corresponding to a response rate of 51.8% (Fig. 1). There were 65 females (55.6%) and 52 males (44.4%), with a mean age of 55.0 ± 16.9 years. All participants were adults attending orthopaedic outpatient clinics at a tertiary referral center.

### Awareness and exposure

Overall, 45 patients (38.5%) reported that they had previously heard of robotic-assisted joint replacement, while 71 (60.7%) had not. The majority of respondents (87.2%, n = 102) stated that they would wish to be informed beforehand if a robot were to be involved in their procedure. Forty-two patients (35.9%) believed that robotic-assisted surgery required additional or longer incisions.

### Attitudes and preferences

Thirteen patients (11.1%) indicated that they would be willing to swap their surgeon for one using robotic-assisted techniques, while 100 (85.5%) would not. On a 0–10 scale, where higher values represented a perception of superior outcomes, the mean rating for robotic vs conventional surgery was 5.6 ± 2.1. The perceived independence of the robot, where 0 indicated full surgeon control and 10 indicated complete autonomy, had a mean score of 4.3 ± 2.7.

### Perceived risks and benefits

When asked about specific aspects of robotic-assisted surgery compared with conventional techniques, participants gave mean scores of 5.2 ± 2.4 for invasiveness, 5.3 ± 2.3 for risk, 5.9 ± 2.2 for operative time, 6.5 ± 2.1 for cost, 5.3 ± 1.7 for postoperative pain, and 5.6 ± 1.9 for recovery time (Table 1).

**Table 1:** Participant demographics and baseline perceptions.

Variable	Mean ± SD or n (%)
Age (y)	55.0 ± 16.9 (range, 18–86)
Gender	Male: 52 (44.4%) female: 65 (55.6%)
Heard of robotic surgery	Yes: 45 (38.5%) no: 71 (60.7%)
Would want to be informed if robot used	102 (87.2%)
Believe robotic surgery involves extra/longer cuts	42 (35.9%)
Would change surgeon for robotic surgery	13 (11.1%)
Perceived outcomes (0–10)	5.6 ± 2.1
Robot independence (0–10)	4.3 ± 2.7
Invasiveness (0–10)	5.2 ± 2.4
Risk (0–10)	5.3 ± 2.3
Operative time (0–10)	5.9 ± 2.2
Cost (0–10)	6.5 ± 2.1
Postoperative pain (0–10)	5.3 ± 1.7
Recovery time (0–10)	5.6 ± 1.9

SD, standard deviation.

### Comparison by awareness of robotic surgery

Of the 117 respondents, 116 provided clear responses regarding awareness of robotic-assisted surgery. Forty-five (38.8%) had heard of it and 71 (61.2%) had not. Participants who were aware of robotics rated the perceived effectiveness of robotic vs conventional surgery significantly higher (6.5 ± 1.8 vs 5.0 ± 2.1, *P* < .001; Welch’s *t*-test) and anticipated lower postoperative pain (4.8 ± 1.5 vs 5.7 ± 1.8, *P* = .006). They also demonstrated lower perceived risk (4.7 ± 2.1 vs 5.8 ± 2.3, *P* = .019). No significant differences were observed between groups for perceptions of invasiveness (*P* = .835), operative time (*P* = .227), cost (*P* = .312), recovery time (*P* = .195), or perceived robot independence (*P* = .319).

Regarding willingness to swap surgeons with respect to awareness of robotics, 10 of 45 respondents (22.2%) who had heard of robotic surgery answered “yes,” compared with 3 of 71 (4.2%) who had not heard of it (χ^2^ = 7.96, *P* = .005) (Table 2).

**Table 2:** Perceptions by awareness of robotic-assisted surgery.

Variable	Heard of robots (n = 45) mean ± SD	Not heard (n = 71) mean ± SD	*P* value
Perceived outcomes (0–10)	6.5 ± 1.8	5.0 ± 2.1	<.001
Robot independence (0–10)	4.6 ± 2.8	4.1 ± 2.6	.319
Invasiveness (0–10)	5.1 ± 2.3	5.2 ± 2.4	.835
Risk (0–10)	4.7 ± 2.1	5.8 ± 2.3	.019
Operative time (0–10)	6.1 ± 2.0	5.7 ± 2.3	.227
Cost (0–10)	6.3 ± 2.2	6.6 ± 2.0	.312
Postoperative pain (0–10)	4.8 ± 1.5	5.7 ± 1.8	.006
Recovery time (0–10)	5.8 ± 1.7	5.4 ± 2.0	.195
Would change surgeon for robotic surgery (yes)	10 (22.2%)	3 (4.2%)	.005

SD, standard deviation.

### Comparison by age group

Among 117 participants, 65 (55.6%) were aged under 60 years and 52 (44.4%) were aged 60 years or older. Awareness of robotic-assisted surgery did not differ significantly between age groups (χ^2^ = 0.26, *P* = .611). Similarly, willingness to swap surgeons did not vary by age (χ^2^ = 0.55, *P* = .457).

Mean ratings were similar across most perception measures. On a 0–10 scale, patients under 60 rated the expected effectiveness of robotic-assisted vs conventional surgery at 5.6 ± 1.9, compared with 5.7 ± 2.3 among those aged 60 or older (*P* = .748). Perceived robot independence was 4.5 ± 2.6 for the younger group and 4.0 ± 2.9 for the older group (*P* = .389). Mean scores for invasiveness (5.2 ± 2.3 vs 5.2 ± 2.6; *P* = .904), risk (5.2 ± 1.9 vs 5.5 ± 2.7; *P* = .563), operative time (5.8 ± 2.1 vs 6.1 ± 2.4; *P* = .466), and recovery time (5.5 ± 1.8 vs 5.7 ± 2.1; *P* = .560) showed no significant differences. Older respondents rated perceived cost slightly higher (6.9 ± 2.0 vs 6.2 ± 2.1), although this difference did not reach statistical significance (*P* = .083). Postoperative pain scores were identical between groups (5.3 ± 1.5 vs 5.3 ± 2.0; *P* = .962) (Table 3).

**Table 3:** Perceptions by age group.

Variable	<60 years (n = 65) mean ± SD	≥60 years (n = 52) mean ± SD	*P* value
Perceived outcomes (0–10)	5.6 ± 1.9	5.7 ± 2.3	.748
Robot independence (0–10)	4.5 ± 2.6	4.0 ± 2.9	.389
Invasiveness (0–10)	5.2 ± 2.3	5.2 ± 2.6	.904
Risk (0–10)	5.2 ± 1.9	5.5 ± 2.7	.563
Operative time (0–10)	5.8 ± 2.1	6.1 ± 2.4	.466
Cost (0–10)	6.2 ± 2.1	6.9 ± 2.0	.083
Postoperative pain (0–10)	5.3 ± 1.5	5.3 ± 2.0	.962
Recovery time (0–10)	5.5 ± 1.8	5.7 ± 2.1	.560
Heard of robotic surgery (yes)	23 (23/64)	22 (22/52)	.611
Would change surgeon for robotic surgery (yes)	9 (13.8 %)	4 (7.7 %)	.457

SD, standard deviation.

## Discussion

This single-center survey provides the first Irish data on patient perceptions of robotic-assisted hip and knee arthroplasty. Awareness of robotic surgery in our cohort was modest, with only 38.5% of patients reporting that they had heard of the technology. Despite this, the majority expressed a desire to be informed if a robot were used during their operation. Those who had previously heard of robotic surgery demonstrated more optimistic expectations, including higher perceived success rates and lower anticipated pain and risk. These findings mirror international trends, where familiarity with robotics through education or media exposure is associated with more favorable attitudes [[11], [12], [13]].

The lower awareness observed in this Irish cohort compared to reports from the United States may reflect differences in exposure to direct-to-consumer marketing and public promotion of robotic technologies. Greater visibility of robotic platforms in the United States may contribute to higher patient familiarity and more technology-driven expectations, whereas the Irish setting may represent a less commercially influenced baseline of patient perception. Despite this, willingness to change surgeons remained low, suggesting that increased awareness does not necessarily translate into changes in patient decision-making.

### Principal findings in context

Our awareness rate aligns with studies from comparable populations, which have shown variable understanding of robotic surgery and frequent misconceptions about robot autonomy. Al Dihan et al. reported that 70.7% of surgical clinic attendees were unaware of robotic surgery and that many believed robots could operate independently [14]. In our cohort, “robot independence” scores were moderate, suggesting that patients generally recognized that the surgeon remains in control.

Patients who had prior awareness of robotics in our study anticipated less postoperative pain, faster recovery, and improved outcomes, consistent with international surveys showing that awareness and interest correlate with optimism about accuracy and outcomes, albeit tempered by concerns regarding cost and operative time [12]. Public perception data reinforce this expectation–evidence gap. Pagani et al. found that two-thirds of respondents believed robotic surgery achieved superior outcomes, and nearly half preferred a low-volume surgeon using a robot to a high-volume surgeon without one, emphasizing the influence of marketing and the need to center patient discussions on surgeon experience and evidence [11]. This highlights the importance of clearly establishing patient expectations during informed consenting processes.

Of interest, no significant differences were observed between younger (<60 years) and older (≥60 years) participants across perception domains. This suggests that age does not independently influence attitudes, supporting previous findings that education and awareness are more influential than demographic factors [12,13].

Although many patients expressed curiosity about robotics, most stated that they would not change surgeons solely to access robotic technology, reinforcing that trust in the surgeon remains the primary determinant of confidence. Similar patterns have been described internationally, where patients regard robotics as an enhancement to, rather than a replacement for, surgical expertise. A large recent study of over 400 arthroplasty patients found similarly cautious attitudes, with most respondents uncertain of the benefits of robotic joint replacement, over half unwilling to accept longer incisions, and only 2.3% actively choosing a surgeon because they offered robotic-assisted procedures [11,14].

### Education, media influence, and modifiable misconceptions

Mass media continues to play a major role in shaping public understanding of new technologies and remains a source of misinformation [15]. Targeted educational interventions can address misconceptions regarding safety, surgeon control, and likely benefits. In a prospective survey of a public open-day demonstration, Fumagalli et al. found that reassurance and willingness to choose robotic surgery improved significantly after participants observed a live robotic demonstration [16]. These findings support the development of concise, evidence-based patient information and visual resources that can be incorporated into preoperative counseling in orthopedic clinics.

High-quality randomized controlled trials have not demonstrated functional or survivorship advantages for robotic-assisted total knee arthroplasty compared with conventional techniques at 10-year follow-up [17]. Systematic reviews continue to report mixed outcomes, noting accuracy gains but also higher cost, increased operative time, and a learning curve for surgeons [18]. Patients should understand that robotic systems are surgeon-controlled, that short-term improvements in precision may not translate to long-term superiority, and that evidence remains mixed.

In Ireland, healthcare is delivered through a mixed public and private system, and access to robotic-assisted surgery remains limited and unevenly distributed across centers. National data suggest that robotic platforms are present in a small number of institutions, with greater availability in private hospitals, raising potential issues of equity of access [19]. This may influence patient awareness and exposure to robotic platforms and should be considered when interpreting these findings. As expansion into the public system begins, transparent communication about selection criteria, surgeon training, and access will be crucial to ensure equitable adoption and maintain public confidence [13].

While patients’ interest in robotics is clear, the data also indicate that many remain unaware of the technology’s current availability or limitations. Educating both patients and referring clinicians will therefore be essential for balanced expectations and appropriate utilization.

### Strengths and limitations

The strengths of this study include its prospective design, consecutive sampling of real-world orthopaedic clinic patients, and alignment of survey content with previously validated constructs from international studies.

However, several limitations must be acknowledged. This was a single-center study with a modest sample size, which may limit external validity. The cross-sectional survey design captures perceptions at a single point in time and cannot determine whether expectations influence postoperative satisfaction or outcomes. Although a response rate was derived through retrospective review of clinic attendance records, the exact number of patients approached or declining participation was not prospectively recorded. Nonresponse bias remains possible, as patients who chose to participate may differ systematically from those who did not respond, which may introduce selection bias and limit the generalizability of findings. Survey response rates in orthopaedic studies are often variable and can be low, which may introduce nonresponse bias and limit the generalizability of findings. [20]

Although the questionnaire was designed based on existing instruments, it is not formally validated. Educational level and digital literacy were not assessed, which may influence comprehension and awareness of robotics. The measure of “awareness” in this study was based on whether participants had previously heard of robotic surgery and does not necessarily reflect accurate knowledge or understanding of the technology, which may influence interpretation of these findings. Some categorical comparisons involved small cell counts, and alternative statistical approaches such as Fisher’s exact test may have been more appropriate. In addition, multiple subgroup comparisons were performed without formal adjustment for multiple testing. These findings should therefore be interpreted with caution.

The study population consisted of a heterogeneous outpatient cohort, likely including patients at different stages of the treatment pathway; however, detailed subgroup data regarding prior arthroplasty status or treatment stage were not specifically recorded, which may influence patient awareness and perceptions.

The sample included patients at different stages of the arthroplasty pathway, and not all respondents were candidates for joint replacement; attitudes among patients actively considering surgery may differ. Finally, perceptions observed in an Irish public-funded context may not fully generalize to systems where access to robotic technology is more widespread or determined by insurance status.

### Future work

Future research should focus on evaluating the impact of structured educational interventions—such as standardized leaflets or short videos—on patient understanding, expectations, and satisfaction. Studies should also explore whether preoperative expectations predict postoperative outcomes or satisfaction scores. Incorporating cost-effectiveness and equity analyses will be important to ensure robotic-assisted arthroplasty is implemented within a value-based framework.

## Conclusions

Limited baseline awareness and mixed attitudes toward robotic-assisted arthroplasty were evident from this study in an Irish healthcare setting. Prior awareness was associated with greater optimism and lower perceived pain and risk, while trust in the surgeon remained the most influential factor in decision-making. As robotic systems become more widely introduced across Irish hospitals, aligning patient education with the evidence base and maintaining transparent access and training policies will be essential to achieving patient-centered, value-conscious adoption

## Ethical approval

This study involved an anonymous, voluntary survey of patients. Informed consent was implied by completion of the questionnaire. No identifiable data were collected.

## CRediT authorship contribution statement

**Conor Farrell:** Writing – original draft, Visualization, Software, Resources, Methodology, Investigation, Formal analysis, Data curation. **Orla Hennessy:** Writing – review & editing, Conceptualization. **Dunamis Akinyemi:** Data curation. **Angela Faustino:** Data curation, Conceptualization. **Fiachra Rowan:** Writing – review & editing, Supervision, Project administration, Conceptualization. **May S. Cleary:** Writing – review & editing, Supervision, Project administration, Conceptualization.

## Conflicts of interest

The authors declare there are no conflicts of interest.

For full disclosure statements refer to https://doi.org/10.1016/j.artd.2026.102062.

## Appendix

Opinion questionnaire about robot-assisted joint replacement

Age: _________ Gender: __________

1. Have you heard of the involvement of robots in knee/hip replacement surgery?

Yes No

2. Would you prefer to know beforehand if a robot is being used during your surgery?

Yes No

3. Would you accept a robot-assisted surgery if it involved extra or longer skin cuts?

Yes No

4. Would you change your surgeon in order to get a robot-assisted knee/hip replacement?

Yes No

5. How do you think robot-assisted joint replacement compares to regular joint replacement?

0 ─── 1 ─── 2 ─── 3 ─── 4 ─── 5 ─── 6 ─── 7 ─── 8 ─── 9 ─── 10Very bad results (0) Excellent results (10)

6. How much independence do you believe the robot has during the surgery?

0 ─── 1 ─── 2 ─── 3 ─── 4 ─── 5 ─── 6 ─── 7 ─── 8 ─── 9 ─── 10Fully controlled by a surgeon (0) Fully independent (10)

7. How invasive do you consider robotic surgery to be?

0 ─── 1 ─── 2 ─── 3 ─── 4 ─── 5 ─── 6 ─── 7 ─── 8 ─── 9 ─── 10Not invasive/Minimally (0) Very invasive/Fully open (10)

8. What risk do you think robotic-assisted surgery carries, compared to regular joint replacement?

0 ─── 1 ─── 2 ─── 3 ─── 4 ─── 5 ─── 6 ─── 7 ─── 8 ─── 9 ─── 10No risk (0) Very risky (10)

9. How long do you believe robotic-assisted surgery takes, compared to regular joint replacement?

0 ─── 1 ─── 2 ─── 3 ─── 4 ─── 5 ─── 6 ─── 7 ─── 8 ─── 9 ─── 10Much slower (0) Much faster (10)

10. What is your opinion on the cost of robotic-assisted surgery, compared to regular joint replacement?

0 ─── 1 ─── 2 ─── 3 ─── 4 ─── 5 ─── 6 ─── 7 ─── 8 ─── 9 ─── 10Very cheap (0) Very expensive (10)

11. How do you think pain following robotic-assisted surgery compares to regular joint replacement?

0 ─── 1 ─── 2 ─── 3 ─── 4 ─── 5 ─── 6 ─── 7 ─── 8 ─── 9 ─── 10No pain (0) Severe pain (10)

12. How do you think recovery time following robotic-assisted surgery compares to regular joint replacement?

0 ─── 1 ─── 2 ─── 3 ─── 4 ─── 5 ─── 6 ─── 7 ─── 8 ─── 9 ─── 10Much slower (0) Much faster (10)

## Appendix A Supplementary data

Conflict of Interest Statement for Akinyemi

Conflict of Interest Statement for Rowan

Conflict of Interest Statement for Cleary

Conflict of Interest Statement for Hennessy

Conflict of Interest Statement for Farrell

Conflict of Interest Statement for Faustino
